# Supplementary material for: Rubinstein-Taybi Syndrome: A Model of Epigenetic Disorder
Source: Genes (Basel). 2021 Jun 24;12(7):968. doi: 10.3390/genes12070968 (PMC8303114; doi:10.3390/genes12070968)
Supplement: Supplementary file 1 [file genes-12-00968-s001.zip › genes-1248377-supplementary.pdf]

Table S1: All of 445 *CREBBP* mutations causing Rubinstein-Taybi syndrome type 1 listed in HGMDPro variant database and reported in the literature (called the 04/27/2021)

Missense/nonsense: 152 mutations

Nucleotide	Protein	Reference
c.40A>G	p.(Arg14Gly)	Bentivegna (2006) BMC Med Genet **7,** 77
c.68C>A	p.(Ser23*)	Coupry (2002) J Med Genet **39,** 415
c.223C>T	p.(Arg75*)	Spena (2015) Clin Genet **88,** 431<br>Kosaki (2020) *Am J Med Genet A* **182:** 1601
c.304C>T	p.(Gln102*)	Roelfsema (2005) Am J Hum Genet **76,** 572
c.376G>T	p.(Gly126*)	Cross (2020) Am J Med Genet A **182,** 2508
c.406C>T	p.(Gln136*)	Petrij (1995) Nature **376,** 348
c.472C>T	p.(Gln158*)	Spena (2015) Clin Genet **88,** 431
c.547C>T	p.(Gln183*)	Spena (2015) Clin Genet **88,** 431
c.613C>T	p.(Gln205*)	Cross (2020) Am J Med Genet A **182,** 2508
c.778C>T	p.(Gln260*)	Balci (2010) Am J Med Genet A **152A,** 1036
c.992C>G	p.(Ser331*)	Pérez-Grijalba (2019) Mol Genet Genomic Med **7,**
c.997G>T	p.(Gly333*)	Cross (2020) Am J Med Genet A **182,** 2508
c.1066C>T	p.(Gln356*)	Kamenarova (2016) Hum Pathol **47,** 144
c.1069C>T	p.(Gln357*)	Petrij (1995) Nature **376,** 348<br>Xiong (2015) *Science* **347:** 1254806<br>Cross (2020) *Am J Med Genet A* **182:** 2508
c.1108C>T	p.(Arg370*)	Coupry (2002) J Med Genet **39,** 415<br>Xiong (2015) *Science* **347:** 1254806<br>Cross (2020) *Am J Med Genet A* **182:** 2508
c.1156C>T	p.(Arg386*)	Cross (2020) Am J Med Genet A **182,** 2508
c.1217T>G	p.(Val406Gly)	Sharma (2010) J Biosci **35,** 187
c.1237C>T	p.(Arg413*)	Coupry (2002) J Med Genet **39,** 415<br>Xiong (2015) *Science* **347:** 1254806<br>Cross (2020) *Am J Med Genet A* **182:** 2508
c.1270C>T	p.(Arg424*)	Bartsch (2005) Hum Genet **117,** 485<br>Xiong (2015) *Science* **347:** 1254806<br>Cross (2020) *Am J Med Genet A* **182:** 2508
c.1279T>G	p.(Cys427Gly)	Sharma (2010) J Biosci **35,** 187
c.1305T>G	p.(Asn435Lys)	Sharma (2010) J Biosci **35,** 187
c.1318C>T	p.(Arg440*)	Pérez-Grijalba (2019) Mol Genet Genomic Med **7,**
c.1447C>T	p.(Arg483*)	Pérez-Grijalba (2019) Mol Genet Genomic Med **7,**
c.1483C>T	p.(Gln495*)	Spena (2015) Clin Genet **88,** 431<br>Cross (2020) *Am J Med Genet A* **182:** 2508
c.1549C>T	p.(Gln517*)	Cross (2020) Am J Med Genet A **182,** 2508
c.1646C>G	p.(Ser549*)	Cross (2020) Am J Med Genet A **182,** 2508
c.1652T>C	p.(Leu551Pro)	Schorry (2008) Am J Med Genet A **146A,**2512
c.1775G>A	p.(Trp592*)	Hu (2018) Genet Med **20,** 1045
c.1801C>T	p.(Arg601Trp)	Cross (2020) Am J Med Genet A **182,** 2508
c.1811T>C	p.(Leu604Pro)	Suzuki (2013) Clin Genet **83,** 291
c.1828C>T	p.(Gln610*)	Schorry (2008) Am J Med Genet A **146A,**2512<br>Xiong (2015) *Science* **347:** 1254806
c.1949A>T	p.(Tyr650Phe)	Spena (2015) Clin Genet **88,** 431
c.1984C>T	p.(Gln662*)	Bentivegna (2006) BMC Med Genet **7,** 77<br>Xiong (2015) *Science* **347:** 1254806
c.2026C>T	p.(Gln676*)	Schorry (2008) Am J Med Genet A **146A,**2512<br>Xiong (2015) *Science* **347:** 1254806
c.2254C>T	p.(Gln752*)	Saettini (2020) J Clin Immunol **40,** 851
c.2302C>T	p.(Arg768*)	Schorry (2008) Am J Med Genet A **146A,**2512<br>Xiong (2015) *Science* **347:** 1254806
c.2461C>T	p.(Gln821*)	Spena (2015) Clin Genet **88,** 431
c.2641C>T	p.(Gln881*)	Pérez-Grijalba (2019) Mol Genet Genomic Med **7,**
c.2678C>T	p.(Ser893Leu)	Schorry (2008) Am J Med Genet A **146A,**2512<br>Bodian (2014) *PLoS One* **9:** e94554<br>Rego (2018) *Cold Spring Harb Mol Case Stud* **4:**
c.2773C>T	p.(Gln925*)	Cross (2020) Am J Med Genet A **182,** 2508

c.2842C>T	p.(Gln948*)	Wieczorek (2009) Am J Med Genet A **149A,**2849<br>Xiong (2015) *Science* **347:** 1254806
c.2911A>T	p.(Arg971*)	Cross (2020) Am J Med Genet A **182,** 2508
c.2941G>A	p.(Ala981Thr)	Coupry (2002) J Med Genet **39,** 415<br>Bodian (2014) *PLoS One* **9:** e94554
c.2959C>T	p.(Gln987*)	Van-Gils (2019) Clin Genet **95,** 420
c.2986G>T	p.(Glu996*)	Bartsch (2005) Hum Genet **117,** 485<br>Xiong (2015) *Science* **347:** 1254806
c.3058G>T	p.(Glu1020*)	Cross (2020) Am J Med Genet A **182,** 2508
c.3097A>T	p.(Lys1033*)	Cross (2020) Am J Med Genet A **182,** 2508
c.3121C>T	p.(Gln1041*)	Lee (2015) Brain Dev **37,** 402
c.3306T>A	p.(Tyr1102*)	Hu (2020) Front Genet **11,** 00473
c.3307C>T	p.(Arg1103*)	Lee (2015) Brain Dev **37,** 402
c.3452G>A	p.(Trp1151*)	Wincent (2016) Mol Genet Genomic Med **4,**39
c.3459C>G	p.(Tyr1153*)	Yu (2019) Mol Genet Genomic Med **7,**
c.3474G>A	p.(Trp1158*)	Bedeschi (2014) Am J Med Genet A **164,**2663
c.3485A>G	p.(Asn1162Ser)	Schorry (2008) Am J Med Genet A **146A,**2512
c.3494G>A	p.(Trp1165*)	Spena (2015) Clin Genet **88,** 431
c.3503A>G	p.(Asn1168Ser)	Wang (2019) Clin Exp Dermatol **44,** e205
c.3514T>C	p.(Ser1172Pro)	Saettini (2020) J Clin Immunol **40,** 851
c.3517C>T	p.(Arg1173*)	Bentivegna (2006) BMC Med Genet **7,** 77<br>Xiong (2015) *Science* **347:** 1254806
c.3524A>G	p.(Tyr1175Cys)	Bartsch (2002) J Med Genet **39,** 496<br>Cross (2020) *Am J Med Genet A* **182:** 2508
c.3535A>G	p.(Ser1179Gly)	Zhang (2014) Zhonghua Er Ke Za Zhi **52,**673<br>Cross (2020) *Am J Med Genet A* **182:** 2508
c.3625C>T	p.(Gln1209*)	Cross (2020) Am J Med Genet A **182,** 2508
c.3639C>A	p.(Cys1213*)	Bartsch (2005) Hum Genet **117,** 485
c.3649C>T	p.(Gln1217*)	Saettini (2020) J Clin Immunol **40,** 851<br>Wei (2021) *Arch Dis Child* **106:** 38
c.3690T>G	p.(Tyr1230*)	Cross (2020) Am J Med Genet A **182,** 2508
c.3719G>A	p.(Cys1240Tyr)	Cross (2020) Am J Med Genet A **182,** 2508
c.3805A>T	p.(Lys1269*)	Coupry (2002) J Med Genet **39,** 415<br>Xiong (2015) *Science* **347:** 1254806
c.3833A>C	p.(Glu1278Ala)	Spena (2015) Clin Genet **88,** 431
c.3833A>G	p.(Glu1278Gly)	Udaka (2005) Congenit Anom (Kyoto) **45,**125
c.3832G>A	p.(Glu1278Lys)	Kalkhoven (2003) Hum Mol Genet **12,** 441<br>Cross (2020) *Am J Med Genet A* **182:** 2508
c.3872A>G	p.(His1291Arg)	Suzuki (2013) Clin Genet **83,** 291
c.4001T>C	p.(Leu1334Pro)	Schorry (2008) Am J Med Genet A **146A,**2512
c.4003G>C	p.(Gly1335Arg)	Li (2010) Am J Med Genet A **152A,** 2939
c.4014G>C	p.(Leu1338Phe)	Lopez-Atalaya (2012) J Med Genet **49,** 66
c.4021C>T	p.(Arg1341*)	Suzuki (2013) Clin Genet **83,** 291
c.4040G>C	p.(Arg1347Pro)	Cross (2020) Am J Med Genet A **182,** 2508
c.4078C>T	p.(Arg1360*)	Chiang (2009) Am J Med Genet A **149A,**1463<br>Xiong (2015) *Science* **347:** 1254806<br>Cross (2020) *Am J Med Genet A* **182:** 2508
c.4112T>A	p.(Val1371Asp)	Yu (2019) Mol Genet Genomic Med **7,**
c.4118C>A	p.(Pro1373His)	Yu (2019) Mol Genet Genomic Med **7,**
c.4133G>C	p.(Arg1378Pro)	Murata (2001) Hum Mol Genet **10,** 1071<br>Xiong (2015) *Science* **347:** 1254806
c.4145C>T	p.(Ser1382Phe)	Cross (2020) Am J Med Genet A **182,** 2508
c.4151A>G	p.(Glu1384Gly)	Sharma (2010) J Biosci **35,** 187
c.4175G>T	p.(Arg1392Leu)	Cross (2020) Am J Med Genet A **182,** 2508
c.4174C>T	p.(Arg1392*)	Schorry (2008) Am J Med Genet A **146A,**2512<br>Xiong (2015) *Science* **347:** 1254806<br>Cross (2020) *Am J Med Genet A* **182:** 2508
c.4216G>T	p.(Asp1406Tyr)	Spena (2015) Clin Genet **88,** 431
c.4224C>A	p.(Cys1408*)	Schorry (2008) Am J Med Genet A **146A,**2512<br>Xiong (2015) *Science* **347:** 1254806
c.4238A>C	p.(His1413Pro)	Udaka (2005) Congenit Anom (Kyoto) **45,**125
c.4244A>G	p.(Gln1415Arg)	Pérez-Grijalba (2019) Mol Genet Genomic Med **7,**

c.4244A>C	p.(Gln1415Pro)	Spena (2015) Clin Genet **88,** 431
c.4243C>T	p.(Gln1415*)	Cross (2020) Am J Med Genet A **182,** 2508
c.4262G>T	p.(Cys1421Phe)	Miller (2020) Cold Spring Harb Mol Case Stud **6,**
c.4277C>G	p.(Thr1426Arg)	Cross (2020) Am J Med Genet A **182,** 2508
c.4280G>C	p.(Arg1427Thr)	Schorry (2008) Am J Med Genet A **146A,**2512
c.4283G>C	p.(Arg1428Pro)	Cross (2020) Am J Med Genet A **182,** 2508
c.4290C>A	p.(Tyr1430*)	Cross (2020) Am J Med Genet A **182,** 2508
c.4297T>C	p.(Tyr1433His)	Cross (2020) Am J Med Genet A **182,** 2508
c.4303G>T	p.(Asp1435Tyr)	Cross (2020) Am J Med Genet A **182,** 2508
c.4304A>T	p.(Asp1435Val)	Bartsch (2005) Hum Genet **117,** 485
c.4340C>T	p.(Thr1447Ile)	Roelfsema (2005) Am J Hum Genet **76,** 572
c.4348T>C	p.(Tyr1450His)	Roelfsema (2005) Am J Hum Genet **76,** 572
c.4361T>A	p.(Leu1454His)	Tajir (2013) Gene **518,** 476
c.4377G>C	p.(Glu1459Asp)	Sharma (2010) J Biosci **35,** 187
c.4394G>A	p.(Gly1465Glu)	Pérez-Grijalba (2019) Mol Genet Genomic Med **7,**
c.4398T>A	p.(Tyr1466*)	Coupry (2002) J Med Genet **39,** 415<br>Xiong (2015) *Science* **347:** 1254806
c.4409A>G	p.(His1470Arg)	Roelfsema (2005) Am J Hum Genet **76,** 572
c.4417G>A	p.(Ala1473Thr)	Cross (2020) Am J Med Genet A **182,** 2508
c.4423C>A	p.(Pro1475Thr)	Spena (2015) Clin Genet **88,** 431
c.4435G>T	p.(Gly1479*)	Bentivegna (2006) BMC Med Genet **7,** 77<br>Xiong (2015) *Science* **347:** 1254806
c.4439A>G	p.(Asp1480Gly)	Liu (2019) N Engl J Med **380,** 2478<br>Cross (2020) *Am J Med Genet A* **182:** 2508
c.4444T>G	p.(Tyr1482Asp)	Lopez-Atalaya (2012) J Med Genet **49,** 66
c.4445A>G	p.(Tyr1482Cys)	Bentivegna (2006) BMC Med Genet **7,** 77
c.4460A>G	p.(His1487Arg)	Cross (2020) Am J Med Genet A **182,** 2508
c.4459C>T	p.(His1487Tyr)	Cross (2020) Am J Med Genet A **182,** 2508
c.4492C>T	p.(Arg1498*)	Coupry (2002) J Med Genet **39,** 415<br>Xiong (2015) *Science* **347:** 1254806<br>Anazi (2018) *Hum Genet* **137:** 105
c.4499A>C	p.(Gln1500Pro)	Schorry (2008) Am J Med Genet A **146A,**2512
c.4508A>G	p.(Tyr1503Cys)	Schorry (2008) Am J Med Genet A **146A,**2512
c.4507T>C	p.(Tyr1503His)	Huang (2018) Cell Physiol Biochem **49,** 295
c.4508A>T	p.(Tyr1503Phe)	Spena (2015) Clin Genet **88,** 431
c.4520T>C	p.(Leu1507Pro)	Spena (2015) Clin Genet **88,** 431
c.4557C>A	p.(Tyr1519*)	Schorry (2008) Am J Med Genet A **146A,**2512<br>Xiong (2015) *Science* **347:** 1254806
c.4557C>G	p.(Tyr1519*)	Pérez-Grijalba (2019) Mol Genet Genomic Med **7,**
c.4597A>T	p.(Ser1533Cys)	Sharma (2010) J Biosci **35,** 187
c.4613C>G	p.(Pro1538Arg)	Wincent (2016) Mol Genet Genomic Med **4,**39
c.4613C>T	p.(Pro1538Leu)	Cross (2020) Am J Med Genet A **182,** 2508
c.4627G>A	p.(Asp1543Asn)	Spena (2015) Clin Genet **88,** 431
c.4627G>T	p.(Asp1543Tyr)	Bentivegna (2006) BMC Med Genet **7,** 77
c.4660A>T	p.(Lys1554*)	Yu (2019) Mol Genet Genomic Med **7,**
c.4665A>C	p.(Glu1555Asp)	Fokstuen (2016) Hum Genomics **10,** 24
c.4672C>T	p.(Gln1558*)	Kalkhoven (2003) Hum Mol Genet **12,** 441<br>Xiong (2015) *Science* **347:** 1254806
c.4762A>T	p.(Lys1588*)	Spena (2015) Clin Genet **88,** 431
c.4804C>T	p.(Arg1602Cys)	Carli (2013) Birth Defects Res A Clin Mol Teratol **97,** 798
c.4813A>T	p.(Lys1605*)	Waldmüller (2015) Mol Cell Probes **29,** 308
c.4837G>A	p.(Val1613Met)	Sharma (2010) J Biosci **35,** 187
c.4874T>C	p.(Met1625Thr)	Schorry (2008) Am J Med Genet A **146A,**2512
c.4879A>T	p.(Lys1627*)	Roelfsema (2005) Am J Hum Genet **76,** 572<br>Xiong (2015) *Science* **347:** 1254806
c.4990C>T	p.(Arg1664Cys)	Zhu (2020) Front Genet **11,** 565078
c.4991G>A	p.(Arg1664His)	Kalkhoven (2003) Hum Mol Genet **12,** 441<br>Cross (2020) *Am J Med Genet A* **182:** 2508
c.5003T>A	p.(Leu1668His)	Sharma (2010) J Biosci **35,** 187
c.5058G>A	p.(Trp1686*)	Pérez-Grijalba (2019) Mol Genet Genomic Med **7,**
c.5060C>T	p.(Ser1687Phe)	Lopez-Atalaya (2012) J Med Genet **49,** 66

c.5170G>A	p.(Glu1724Lys)	Sharma (2010) J Biosci **35,** 187<br>Angius (2019) *Am J Med Genet A* **179:** 634
c.5204C>G	p.(Thr1735Arg)	Sharma (2010) J Biosci **35,** 187
c.5635C>T	p.(Gln1879*)	Bartsch (2005) Hum Genet **117,** 485
c.5638C>T	p.(Gln1880*)	Monies (2017) Hum Genet **136,** 921<br>Yu (2019) *Mol Genet Genomic Med* **7:** e1009
c.5905C>T	p.(Gln1969*)	Yu (2019) Mol Genet Genomic Med **7,**<br>Cross (2020) *Am J Med Genet A* **182:** 2508
c.5933A>G	p.(Asn1978Ser)	Coupry (2002) J Med Genet **39,** 415<br>Bodian (2014) *PLoS One* **9:** e94554
c.6010C>T	p.(Arg2004*)	Roelfsema (2005) Am J Hum Genet **76,** 572
c.6019C>T	p.(Gln2007*)	Bartsch (2005) Hum Genet **117,** 485
c.6127C>T	p.(Gln2043*)	Coupry (2002) J Med Genet **39,** 415
c.6133C>T	p.(Gln2045*)	Roelfsema (2005) Am J Hum Genet **76,** 572
c.6283C>T	p.(Gln2095*)	Roelfsema (2005) Am J Hum Genet **76,** 572
c.6661A>C	p.(Met2221Leu)	Coupry (2002) J Med Genet **39,** 415
c.6728C>T	p.(Ala2243Val)	Coupry (2002) J Med Genet **39,** 415

Small deletions: 96 mutations

Nucleotide	Protein	Reference
c.173_185del13	p.(Asn58Metfs*25)	Cross (2020) Am J Med Genet A **182,** 2508
c.201_202delTA	p.(His67Glnfs*14)	Qaksen (2009) Genet Couns **20,**255
c.236delG	p.(Gly79Alafs*8)	Roelfsema (2005) Am J Hum Genet **76,** 572
c.474_493del20	p.(Val159Profs*15)	Bartsch (2005) Hum Genet **117,**485
c.494_507del14	p.(Ser165Thrfs*11)	Cross (2020) Am J Med Genet A **182,** 2508
c.537delT	p.(Asn180Thrfs*18)	Schorry (2008) Am J Med Genet A **146A,** 2512
c.547delC	p.(Gln183Argfs*15)	Schorry (2008) Am J Med Genet A **146A,** 2512
c.662_698del	p.(Gly221Alafs*11)	Cross (2020) Am J Med Genet A **182,** 2508
c.806_807delTA	p.(Ile269Asnfs*12)	Schorry (2008) Am J Med Genet A **146A,** 2512
c.810_811delTG	p.(Gly271Glufs*10)	Spena (2015) Clin Genet **88,**431
c.885_886delCC	p.(Gln296Valfs*53)	Spena (2015) Clin Genet **88,**431
c.904_905delAG	p.(Ser302Hisfs*47)	Roelfsema (2005) Am J Hum Genet **76,** 572
c.1044delT	p.(Glu349Lysfs*5)	Welters (2019) Eur J Endocrinol **,**<br>Cross (2020) *Am J Med Genet A***182:** 2508
c.1113delG	p.(Glu371Aspfs*18)	Gao (2018) Zhonghua Yi Xue Za Zhi **98,** 3426
c.1124delG	p.(Gly375Glufs*14)	Cross (2020) Am J Med Genet A **182,** 2508
c.1225_1231delTGTGCAT	p.(Cys409Leufs*23)	Cross (2020) Am J Med Genet A **182,** 2508
c.1238delG	p.(Arg413Hisfs*21)	Schorry (2008) Am J Med Genet A **146A,** 2512
c.1251_1252delTC	p.(His418Leufs*8)	Van-Gils (2019) Clin Genet **95,**420
c.1257delG	p.(Trp419*)	Spena (2015) Clin Genet **88,**431
c.1280_1281delGT	p.(Cys427Serfs*11)	Yu (2019) Mol Genet Genomic Med **7,**
c.1382_1389delGCACAGGG	p.(Gly461Alafs*9)	Roelfsema (2005) Am J Hum Genet **76,** 572
c.1425delC	p.(Ile476*)	Schorry (2008) Am J Med Genet A **146A,** 2512
c.1515_1521delTGGCCAG	p.(Gly506Asnfs*11)	Spena (2015) Clin Genet **88,**431
c.1715delG	p.(Gly572Glufs*17)	Tang (2019) Orphanet J Rare Dis **14,** 45
c.1733delC	p.(Pro578Glnfs*11)	Udaka (2005) Congenit Anom (Kyoto) **45,** 125
c.1891_1895delGCCTA	p.(Ala631Cysfs*2)	Bartsch (2005) Hum Genet **117,**485
c.1907_1912delTGGAAG	p.(Val636_Glu637del)	Cross (2020) Am J Med Genet A **182,** 2508
c.1941+1_1941+5delGTAGG	Not yet available	Pérez-Grijalba (2019) Mol Genet Genomic Med **7,**
c.2064_2077del14	p.(Gly689Cysfs*32)	Huh (2015) Ann Clin Lab Sci **45,** 458
c.2199delG	p.(Gln733Hisfs*15)	Yoo (2015) Int J Mol Sci **16,**5697
c.2204delC	p.(Pro735Hisfs*13)	Maddirevula (2018) Genet Med **20,** 12
c.2239_2246delATGAACCA	p.(Met747Leufs*82)	Suzuki (2013) Clin Genet **83,**291
c.2330delG	p.(Gly777Valfs*7)	Cross (2020) Am J Med Genet A **182,** 2508
c.2356delC	p.(Gln786Argfs*21)	Lopez-Atalaya (2012) J Med Genet **49,** 66
c.2459delC	p.(Ser820Tyrfs*29)	Yu (2019) Mol Genet Genomic Med **7,**
c.2479delG	p.(Ala827Leufs*22)	Schorry (2008) Am J Med Genet A **146A,** 2512
c.2527delC	p.(Leu843Tyrfs*6)	Schorry (2008) Am J Med Genet A **146A,** 2512
c.2621delC	p.(Pro874Hisfs*4)	Pérez-Grijalba (2019) Mol Genet Genomic Med **7,**
c.2715_2716delAG	p.(Val906Alafs*63)	Spena (2015) Clin Genet **88,**431
c.2713_2719delTCAGTGC	p.(Ser905Profs*20)	Cross (2020) Am J Med Genet A **182,** 2508
c.2747delC	p.(Pro916Leufs*11)	Schorry (2008) Am J Med Genet A **146A,** 2512

c.2825delC	p.(Pro942Leufs*56)	Van-Gils (2019) Clin Genet **95**,420
c.2827delC	p.(Gln943Serfs*55)	Coupry (2002) J Med Genet **39**,415
c.3032delG	p.(Gly1011Valfs*11)	Suzuki (2013) Clin Genet **83**,291
c.3260delC	p.(Pro1087Glnfs*12)	Schorry (2008) Am J Med Genet A **146A,** 2512
c.3292delC	p.(Leu1098*)	Cross (2020) Am J Med Genet A **182,** 2508
c.3330_3334delTTTCC	p.(Phe1111Alafs*15)	Cross (2020) Am J Med Genet A **182,** 2508
c.3396_3400delCATGG	p.(Met1133Profs*34)	Roelfsema (2005) Am J Hum Genet **76,** 572
c.3432_3433delAG	p.(Gly1145Alafs*23)	Roelfsema (2005) Am J Hum Genet **76,** 572<br>Cross (2020) *Am J Med Genet A***182:** 2508
c.3501_3502delTA	p.(Tyr1167*)	Murata (2001) Hum Mol Genet **10,** 1071
c.3608_3609+5delAGGTACA	Not yet available	Spena (2015) Clin Genet **88**,431
c.3609+2_3609+5delTACA	Not yet available	Pérez-Grijalba (2019) Mol Genet Genomic Med **7,**
c.3661_3665delATTCC	p.(Ile1221Serfs*11)	Cross (2020) Am J Med Genet A **182,** 2508
c.3715_3716delAA	p.(Lys1239Valfs*14)	Bentivegna (2006) BMC Med Genet **7,** 77
c.3751delC	p.(Leu1251Trpfs*25)	Spena (2015) Clin Genet **88**,431
c.3767_3769delCAC	p.(Ser1256*)	Bartsch (2005) Hum Genet **117**,485
c.3832delG	p.(Glu1278Asnfs*35)	Cross (2020) Am J Med Genet A **182,** 2508
c.3858_3859delTG	p.(Cys1286Trpfs*13)	Spena (2015) Clin Genet **88**,431
c.4080_4090del11	p.(Val1361Leufs*23)	Petrij (2000) J Med Genet **37**,168
c.4129_4133+9del14	Not yet available	Kosaki (2020) Am J Med Genet A **182,** 1601
c.4169delC	p.(Pro1390Hisfs*69)	Schorry (2008) Am J Med Genet A **146A,** 2512
c.4189_4192delTTTG	p.(Phe1397Leufs*61)	Lee (2015) Brain Dev **37,** 402
c.4256_4257delCT	p.(Ser1419*)	Kalkhoven (2003) Hum Mol Genet **12,** 441
c.4274delA	p.(Asn1425Thrfs*34)	Van-Gils (2019) Clin Genet **95**,420
c.4399delG	p.(Val1467*)	Roelfsema (2005) Am J Hum Genet **76,** 572
c.4400delT	p.(Val1467Glyfs*83)	Spena (2015) Clin Genet **88**,431
c.4400_4401delTG	p.(Val1467Aspfs*11)	Lee (2015) Brain Dev **37,** 402
c.4404_4405delAG	p.(Gly1469Alafs*9)	Murata (2001) Hum Mol Genet **10,** 1071
c.4425_4426delTC	p.(Pro1476Lysfs*2)	Schorry (2008) Am J Med Genet A **146A,** 2512
c.4444delT	p.(Tyr1482Thrfs*68)	Schorry (2008) Am J Med Genet A **146A,** 2512
c.4611delG	p.(Tyr1539Ilefs*11)	Udaka (2005) Congenit Anom (Kyoto) **45,** 125
c.4644_4645delGT	p.(Leu1549Argfs*5)	Cross (2020) Am J Med Genet A **182,** 2508
c.4650_4654delAGAGA	p.(Glu1551Hisfs*2)	Spena (2015) Clin Genet **88**,431<br>Milani (2016) *Pediatr Blood Cancer* **63:** 572
c.4728+2delT	Not yet available	Cross (2020) Am J Med Genet A **182,** 2508
c.4837delG	p.(Val1613Cysfs*22)	Kalkhoven (2003) Hum Mol Genet **12,** 441
c.4897_4899delTTC	p.(Phe1633del)	Wang (2020) J Mol Neurosci **epub,** epub
c.4945delA	p.(Ile1649Serfs*95)	Coupry (2002) J Med Genet **39**,415
c.4963delC	p.(Leu1655Cysfs*89)	Bentivegna (2006) BMC Med Genet **7,** 77<br>Al-Qattan (2019) *BMC Med Genet* **20:** 12
c.4995_4999delCGCCT	p.(Ala1666Profs*67)	Zhang (2014) Zhonghua Er Ke Za Zhi **52,** 673
c.5016delA	p.(Asp1673Thrfs*71)	Schorry (2008) Am J Med Genet A **146A,** 2512
c.5039_5041delCCT	p.(Ser1680del)	Schorry (2008) Am J Med Genet A **146A,** 2512<br>de Vries (2016) *Eur J Hum Genet* **24:** 1363<br>Van-Gils (2019) *Clin Genet* **95:**420
c.5722delC	p.(Gln1908Serfs*7)	Cross (2020) Am J Med Genet A **182,** 2508
c.5790delC	p.(Thr1931Profs*45)	Spena (2015) Clin Genet **88**,431
c.5837delC	p.(Pro1946Hisfs*30)	Spena (2015) Clin Genet **88**,431<br>Yu (2019) *Mol Genet Genomic Med* **7:** e1009
c.5877_5881delTCGGC	p.(Arg1960Aspfs*4)	Hu (2018) Genet Med **20,** 1045
c.5948delC	p.(Pro1983Glnfs*16)	Lee (2015) Brain Dev **37,** 402
c.5986delG	p.(Ala1996Profs*3)	Cross (2020) Am J Med Genet A **182,** 2508
c.6043delA	p.(Ser2015Alafs*25)	Bentivegna (2006) BMC Med Genet **7,** 77
c.6065_6071delAGCAGGC	p.(Gln2022Argfs*16)	Bentivegna (2006) BMC Med Genet **7,** 77
c.6107_6116del10	p.(Pro2036Argfs*36)	Pérez-Grijalba (2019) Mol Genet Genomic Med **7,**
c.6113_6137del	p.(Pro2038Argfs*29)	Cross (2020) Am J Med Genet A **182,** 2508
c.6122_6125delCCAT	p.(Ser2041Cysfs*33)	Chiang (2009) Am J Med Genet A **149A,** 1463
c.6166delG	p.(Val2056Cysfs*19)	Spena (2015) Clin Genet **88**,431
c.6192delC	p.(Ser2065Alafs*10)	Spena (2015) Clin Genet **88**,431
c.6221_6230del10	p.(Leu2074Profs*11)	Cross (2020) Am J Med Genet A **182,** 2508
c.6250delC	p.(Gln2084Serfs*4)	Spena (2015) Clin Genet **88**,431

Small insertions: 56 mutations

Nucleotide	Protein	Reference
c.134_135insCA	p.(Pro46Asnfs*7)	Schorry (2008) Am J Med Genet A **146A,** 2512
c.243_244insTA	p.(Ile82*)	Cross (2020) Am J Med Genet A **182,** 2508
c.317dupA	p.(Pro107Alafs*5)	Spena (2015) Clin Genet **88,** 431
c.605dupC	p.(Gln203Thrfs*39)	de Kort (2014) Am J Med Genet A **164,** 1332
c.840dupT	p.(Ser281*)	Coupry (2002) J Med Genet **39,** 415
c.967_974dup	p.(Met325Ilefs*32)	Cross (2020) Am J Med Genet A **182,** 2508
c.1062dupA	p.(Gln355Thrfs*12)	Yu (2019) Mol Genet Genomic Med **7,**<br>Wang (2020) *Hum Genet* **139:** 473
c.1481dupA	p.(Asn494Lysfs*34)	Roelfsema (2005) Am J Hum Genet **76,** 572
c.1570dupC	p.(Leu524Profs*4)	Spena (2015) Clin Genet **88,** 431
c.1735dupA	p.(Thr579Asnfs*7)	Roelfsema (2005) Am J Hum Genet **76,** 572
c.1915_1916dupGA	p.(Asp639Glufs*17)	Spena (2015) Clin Genet **88,** 431
c.2041_2042insC	p.(Asn681Thrfs*45)	Pérez-Grijalba (2019) Mol Genet Genomic Med **7,**
c.2045dupA	p.(Pro683Alafs*43)	Coupry (2002) J Med Genet **39,** 415
c.2057dupC	p.(Ala687Serfs*39)	Eser (2017) Turk J Pediatr **59,** 601
c.2178dupC	p.(Met727Hisfs*105)	Schorry (2008) Am J Med Genet A **146A,** 2512
c.2190dupG	p.(Asn731Glufs*101)	Pérez-Grijalba (2019) Mol Genet Genomic Med **7,**
c.2616dup	p.(Thr873Aspfs* 97)	Sequeo (2020) J Med Genet **57,** 760
c.2308_2315dupCCTCAGCC	p.(Pro773Leufs*6)	Jackson (2020) Am J Med Genet C Semin Med Genet **184,** 578
c.2456dup	p.(Ser820Valfs*12)	Cross (2020) Am J Med Genet A **182,** 2508
c.2497dupC	p.(Leu833Profs*137)	Pérez-Grijalba (2019) Mol Genet Genomic Med **7,**
c.2513_2514insGCCA	p.(Gln839Profs*132)	Schorry (2008) Am J Med Genet A **146A,** 2512
c.2749dupA	p.(Thr917Asnfs*53)	Udaka (2005) Congenit Anom (Kyoto) **45,** 125
c.2787dup	p.(Pro930Alafs*40)	Cross (2020) Am J Med Genet A **182,** 2508
c.2810dupC	p.(Ser938Valfs*32)	Wincent (2016) Mol Genet Genomic Med **4,** 39<br>Wincent (2016) *Mol Genet Genomic Med* **4:** 367
c.2854_2863dup	p.(Gln955Argfs*18)	Cross (2020) Am J Med Genet A **182,** 2508
c.2910dup	p.(Arg971Glnfs*21)	Cross (2020) Am J Med Genet A **182,** 2508
c.3020_3021dup	p.(Pro1008Serfs*15)	Cross (2020) Am J Med Genet A **182,** 2508
c.3096dupT	p.(Lys1033*)	Coupry (2002) J Med Genet **39,** 415
c.3168dup	p.(Val1057Serfs*4)	Cross (2020) Am J Med Genet A **182,** 2508
c.3250dupA	Not yet available	Schorry (2008) Am J Med Genet A **146A,** 2512
c.3250+1dupG	Not yet available	Spena (2015) Clin Genet **88,** 431
c.3351_3352dupCC	p.(Gln1118Profs*13)	Bentivegna (2006) BMC Med Genet **7,** 77
c.3475dupC	p.(Leu1159Profs*10)	Schorry (2008) Am J Med Genet A **146A,** 2512
c.3511dupA	p.(Thr1171Asnfs*6)	Schorry (2008) Am J Med Genet A **146A,** 2512
c.3545dupC	p.(Glu1183Argfs*4)	Udaka (2005) Congenit Anom (Kyoto) **45,** 125
c.3546_3547insCC	p.(Glu1183Profs*68)	Marzuillo (2013) BMC Med Genet **14,** 28
c.3824dupT	p.(Leu1275Phefs*8)	Roelfsema (2005) Am J Hum Genet **76,** 572
c.3914+4dupA	Not yet available	Van-Gils (2019) Clin Genet **95,** 420
c.4078dup	p.(Arg1360Profs*28)	Cross (2020) Am J Med Genet A **182,** 2508
c.4268dupC	p.(Pro1424Serfs*13)	Waszak (2018) Lancet Oncol **19,** 785
c.4321dupC	p.(Arg1441Profs*12)	Bartsch (2005) Hum Genet **117,** 485
c.4458_4459insT	p.(His1487Serfs*4)	Cross (2020) Am J Med Genet A **182,** 2508
c.5212_5213insCCTCGGTCCTGCAC	p.(His1738Profs*11)	Murata (2001) Hum Mol Genet **10,**1071
c.4397_4400dupATGT	p.(Thr1468Cysfs*12)	Wincent (2016) Mol Genet Genomic Med **4,** 39
c.4482dupC	p.(Lys1495Glnfs*24)	Lee (2015) Brain Dev **37,** 402
c.4644_4646dupGTT	p.(Leu1549dup)	Herriot (2016) Clin Genet **89,** 355<br>Cross (2020) *Am J Med Genet A***182:** 2508
c.4944dupC	p.(Ile1649Hisfs*11)	Schorry (2008) Am J Med Genet A **146A,** 2512
c.5790dup	p.(Thr1931Hisfs*35)	Cross (2020) Am J Med Genet A **182,** 2508
c.5793dupC	p.(Thr1932Hisfs*34)	Udaka (2005) Congenit Anom (Kyoto) **45,** 125
c.5837dupC	p.(Pro1947Thrfs*19)	Spena (2015) Clin Genet **88,** 431<br>Rokunohe (2016) *J Dermatol Sci* **83:**240<br>Cross (2020) *Am J Med Genet A***182:** 2508
c.5838_5857dup20	p.(Pro1953Hisfs*30)	Spena (2015) Clin Genet **88,** 431<br>Tornese (2015) *Clin Endocrinol (Oxf)* **83:** 437
c.5845dup	p.(Ala1949Glyfs*17)	Cross (2020) Am J Med Genet A **182,** 2508
c.5991dupC	p.(Val1998Argfs*343)	Spena (2015) Clin Genet **88,** 431

c.6044_6050dupGCATGCC	p.(Pro2018Hisfs*325)	Bartsch (2005) Hum Genet **117,** 485
c.6351_6352dupCC	p.(Gln2118Profs*26)	Schorry (2008) Am J Med Genet A **146A,** 2512
c.6113_6137dup	p.(Ala2047CysfsTer29)	Cross (2020) Am J Med Genet A **182,** 2508

Small indels: 7 mutations

Nucleotide	Protein	Reference
c.139delAins13	p.(Asn47delinsSerSer*)	Coupry (2002) J Med Genet **39,** 415
c.1129_1131delGTTinsCAATG	p.(Val377Glnfs*13)	Yu (2019) Mol Genet Genomic Med **7,**
c.2817_2818delinsT	p.(Ala940Leufs*58)	Cross (2020) Am J Med Genet A **182,** 2508
c.2829_2834delGTCATCins11	p.(Gln943Hisfs*57)	Kosaki (2010) Am J Med Genet A **152A,** 1844
c.3369_3369+6delAGTAAGTinsCA	Not yet available	Coupry (2002) J Med Genet **39,** 415
c.3504_3527delins3528_3609+30insA	p.(Asn1168LysfsTer32)	Cross (2020) Am J Med Genet A **182,** 2508
c.3659_3660delinsATGGTA	p.(Thr1220Asnfs*15)	Cross (2020) Am J Med Genet A **182,** 2508

Splicing: 40 mutations

Nucleotide	Reference
c.85+1G>T	Lopez-Atalaya (2012) J Med Genet **49,** 66
c.1216+1G>A	Bartsch (2002) J Med Genet **39,** 496<br>Xiong (2015) *Science* **347:** 1254806
c.1216+2T>A	Cross (2020) Am J Med Genet A **182,** 2508
c.1573+1G>A	Cross (2020) Am J Med Genet A **182,** 2508
c.1676+1G>A	Bartsch (2002) J Med Genet **39,** 496
c.1823+1G>A	Schorry (2008) Am J Med Genet A **146A,** 2512<br>Xiong (2015) *Science* **347:** 1254806
c.1823+5G>A	Spena (2015) Clin Genet **88,** 431<br>Strauss (2018) *Genet Med* **20:** 31<br>Crowgey (2019) *J Mol Diagn* **21:** 687
c.1941+1G>A	Schorry (2008) Am J Med Genet A **146A,** 2512<br>Xiong (2015) *Science* **347:** 1254806
c.1941+1G>T	Yu (2019) Mol Genet Genomic Med **7,**
c.1941+2T>C	Cross (2020) Am J Med Genet A **182,** 2508
c.2158+1G>A	Trujillano (2017) Eur J Hum Genet **25,** 176
c.2881-13G>A	Wincent (2016) Mol Genet Genomic Med **4,** 39<br>Lord (2019) *Genome Res* **29:** 159
c.3060+1G>A	Schorry (2008) Am J Med Genet A **146A,** 2512<br>Xiong (2015) *Science* **347:** 1254806
c.3060+1G>T	Cross (2020) Am J Med Genet A **182,** 2508
c.3369+1G>T	Cross (2020) Am J Med Genet A **182,** 2508
c.3609+1G>T	Pérez-Grijalba (2019) Mol Genet Genomic Med **7,**
c.3698+1G>A	Udaka (2005) Congenit Anom (Kyoto) **45,** 125
c.3698+3A>T	Coupry (2002) J Med Genet **39,** 415
c.3779+1G>A	Dauwerse (2016) Eur J Hum Genet **24,** 1639<br>Cross (2020) *Am J Med Genet A* **182:** 2508
c.3779+1G>T	Li (2017) Zhongguo Dang Dai Er Ke Za Zhi **19,** 1155<br>Cross (2020) *Am J Med Genet A* **182:** 2508
c.3779+2T>C	Dauwerse (2016) Eur J Hum Genet **24,** 1639
c.3779+3A>T	Dauwerse (2016) Eur J Hum Genet **24,** 1639
c.3779+5G>C	Dauwerse (2016) Eur J Hum Genet **24,** 1639
c.3837-2A>C	Van-Gils (2019) Clin Genet **95,** 420
c.3837-2A>G	Schorry (2008) Am J Med Genet A **146A,** 2512<br>Xiong (2015) *Science* **347:** 1254806
c.3837-2A>T	Kalkhoven (2003) Hum Mol Genet **12,** 441<br>Xiong (2015) *Science* **347:** 1254806
c.3836+1G>A	Udaka (2005) Congenit Anom (Kyoto) **45,** 125<br>Xiong (2015) *Science* **347:** 1254806<br>Cross (2020) *Am J Med Genet A* **182:** 2508
c.3836+5G>C	Kalkhoven (2003) Hum Mol Genet **12,** 441<br>Xiong (2015) *Science* **347:** 1254806<br>Wincent (2016) *Mol Genet Genomic Med* **4:** 39
c.3915-1G>A	Roelfsema (2005) Am J Hum Genet **76,** 572
c.3914+1G>A	Cross (2020) Am J Med Genet A **182,** 2508

c.4133+1G>A	Kalkhoven (2003) Hum Mol Genet **12,** 441<br>Xiong (2015) *Science* **347:** 1254806
c.4281-7C>G	Lopez-Atalaya (2012) J Med Genet **49,** 66
c.4280+2T>C	Coupry (2002) J Med Genet **39,** 415<br>Xiong (2015) *Science* **347:** 1254806
c.4394+5G>T	Spena (2015) Clin Genet **88,** 431
c.4561-5C>G	Coupry (2002) J Med Genet **39,** 415
c.4559A>G	Coupry (2002) J Med Genet **39,** 415
c.4560+1G>A	Schorry (2008) Am J Med Genet A **146A,** 2512
c.4728+1G>A	Bentivegna (2006) BMC Med Genet **7,** 77<br>Xiong (2015) *Science* **347:** 1254806
c.4890+1G>A	Cross (2020) Am J Med Genet A **182,** 2508
c.4890+2T>C	Maddirevula (2018) Genet Med **20,** 12

Gross deletions: 89 mutations

Description	Reference
>120 kb ex. 1-31	Bartsch (2002) J Med Genet **39,** 496
>134191bp incl ex. 1-26	Aradhya (2012) Genet Med **14,** 594
>154855bp incl entire gene	Aradhya (2012) Genet Med **14,** 594
>155741bp incl entire gene	Aradhya (2012) Genet Med **14,** 594
>20113bp incl ex. 4-6	Aradhya (2012) Genet Med **14,** 594
>285bp incl ex. 16	Aradhya (2012) Genet Med **14,** 594
>560 kb	Coupry (2002) J Med Genet **39,** 415
>903bp incl ex. 2	Aradhya (2012) Genet Med **14,** 594
>9877bp incl ex. 17-21	Aradhya (2012) Genet Med **14,** 594
~120 kb incl. ex. 6-31 + entire *TRAP1*	Md Ahid (2012) J Med Case Rep **6,** 30
~240kb	Wincent (2016) Mol Genet Genomic Med **4,** 39
0.5-2 kb, ex. 27-28	Boone (2010) Hum Mutat **31,** 1326
0.93-9 kb ex. 22 to IVS23	Rusconi (2015) Hum Genet **134,** 613
1.35-1.38 Mb incl. entire gene +27 others	Rusconi (2015) Hum Genet **134,** 613
1.4 Mb	Alabdullatif (2017) Clin Genet **91,** 616
10,916 bp IVS13-342_IVS16+7306	Udaka (2006) Genet Test **10,** 265
147 kb partial gene	Asadollahi (2014) J Med Genet **51,** 677
148 bp nt. 86	Bartsch (2002) J Med Genet **39,** 496
150 kb incl. entire gene	Gervasini (2007) Genomics **90,** 567
154-164 kb ex. 22-31 +*TRAP1* +*DNASE1* +part *SLX4*	Rusconi (2015) Hum Genet **134,** 613
17-27 kb ex. 1-IVS1	Rusconi (2015) Hum Genet **134,** 613
194-208 kb 5-UTR to ex. 31	Rusconi (2015) Hum Genet **134,** 613
2.6 Mb incl. entire gene	Gervasini (2007) Genomics **90,** 567
210kb incl 5' half of gene	Kosaki (2011) Am J Med Genet A **155,**1189
23 bp, c.6395_6417	Cross (2020) Am J Med Genet A **182,**2508
25 bp, c.6113_6137	Cross (2020) Am J Med Genet A **182,**2508
28-43 kb ex. 4-16	Rusconi (2015) Hum Genet **134,** 613
3' end of gene incl. ex. 17-31	Coupry (2002) J Med Genet **39,** 415
3' gene	Blough (2000) Am J Med Genet **90,** 29
360 kb incl. ex. 1-2	Bourdeaut (2014) Pediatr Blood Cancer **61,** 383
37 bp, c.662_698	Cross (2020) Am J Med Genet A **182,**2508
394-413 kb from IVS2 +7 other genes	Rusconi (2015) Hum Genet **134,** 613
398.43 kb incl. ex. 22-31 +*SLX4* +*DNASE1* +*TRAP1*	Al-Qattan (2020) Case Rep Genet **2020**6143050
480-494 kb incl. entire gene + 7 others	Rusconi (2015) Hum Genet **134,** 613
5-16 kb incl. ex. 31	Rusconi (2015) Hum Genet **134,** 613
5-6 kb incl ex. 27-28	Tsai (2011) Eur J Hum Genet **19,** 43
5' gene	Blough (2000) Am J Med Genet **90,** 29
500 kb incl. entire gene	Gervasini (2007) Genomics **90,** 567
520.7 kb incl. entire gene +*ADCY9* +*SRL*	Wójcik (2010) Am J Med Genet A **152A,** 479
59-64 kb ex. 29-31 to IVS10 *TRAP1*	Rusconi (2015) Hum Genet **134,** 613
6-10 kb IVS11-ex. 15	Rusconi (2015) Hum Genet **134,** 613
61,640 bp IVS3+10556_IVS26+149	Udaka (2006) Genet Test **10,** 265
65-70 kb ex. 12-31 to IVS1 *TRAP1*	Rusconi (2015) Hum Genet **134,** 613
713 bp, ex. 2	Petrij (2000) J Med Genet **37,** 168
741-760 kb incl. entire gene + 9 others	Rusconi (2015) Hum Genet **134,** 613
81.4kb incl ex. 29-31, *DNASE1* and *TRAP1*	Lai (2012) Gene **499,** 182

84-98 kb incl. ex. 1-2	Rusconi (2015) Hum Genet **134,** 613
9-24 kb around ex. 2	Isidor (2010) Am J Med Genet A **152A,**1847
c.4133-154_-2del153	Schorry (2008) Am J Med Genet A **146A,** 2512
codons 267-2443	Bartsch (1999) Eur J Hum Genet **7,** 748
codons 822-2443	Bartsch (1999) Eur J Hum Genet **7,** 748
entire gene	Fokstuen (2016) Hum Genomics **10,** 24
ex. 1	Roelfsema (2005) Am J Hum Genet **76,**572
ex. 1	Balci (2010) Am J Med Genet A **152A,**1036
ex. 1-16	Udaka (2006) Genet Test **10,** 265
ex. 1-19	Roelfsema (2005) Am J Hum Genet **76,**572
ex. 1-2	Roelfsema (2005) Am J Hum Genet **76,**572
ex. 12-31	Yu (2019) Mol Genet Genomic Med **7,**
ex. 13-16	Yu (2019) Mol Genet Genomic Med **7,**
ex. 14-31	Saettini (2020) J Clin Immunol **40,** 851
ex. 17-21	Calì (2013) Genet Mol Res **12,** 2809
ex. 2-3	Saettini (2020) J Clin Immunol **40,** 851
ex. 21	Pérez-Grijalba (2019) Mol Genet Genomic Med **7,**
ex. 21-28	Yu (2019) Mol Genet Genomic Med **7,**
ex. 22-23	Yu (2019) Mol Genet Genomic Med **7,**
ex. 24-31	Pérez-Grijalba (2019) Mol Genet Genomic Med **7,**
ex. 26-30	Pérez-Grijalba (2019) Mol Genet Genomic Med **7,**
ex. 28-31	Zimmermann (2007) Eur J Hum Genet **15,** 837
ex. 29-30	Pérez-Grijalba (2019) Mol Genet Genomic Med **7,**
ex. 29-31	Saettini (2020) J Clin Immunol **40,** 851
ex. 3-31	Pérez-Grijalba (2019) Mol Genet Genomic Med **7,**
ex. 31	Roelfsema (2005) Am J Hum Genet **76,**572
ex. 5-31	Udaka (2006) Genet Test **10,** 265
ex. 6-31	Lee (2015) Brain Dev **37,** 402
incl. entire gene	Blough (2000) Am J Med Genet **90,** 29
incl. entire gene + *TRAP1* and *ADCY9*	Wu (2020) Orphanet J Rare Dis **15,** 101
incl. ex. 12	Coupry (2004) Hum Mutat **23,** 278
incl. ex. 17-28	Cross (2020) Am J Med Genet A **182,**2508
incl. ex. 2	Coupry (2004) Hum Mutat **23,** 278
incl. ex. 2-3	Cross (2020) Am J Med Genet A **182,**2508
incl. ex. 24-28	Cross (2020) Am J Med Genet A **182,**2508
incl. ex. 27-29	Cross (2020) Am J Med Genet A **182,**2508
incl. ex. 29-31	Cross (2020) Am J Med Genet A **182,**2508
incl. ex. 30	Coupry (2004) Hum Mutat **23,** 278
incl. ex. 6-13	Cross (2020) Am J Med Genet A **182,**2508
incl. ex. 7-8	Cross (2020) Am J Med Genet A **182,**2508
incl. ex. 9-14	Cross (2020) Am J Med Genet A **182,**2508
incl. mid-3' gene	Blough (2000) Am J Med Genet **90,** 29
N-*inal region of gene	Coupry (2002) J Med Genet **39,** 415

Gross insertions: 2 mutations

Description	Reference
ex. 1	Roelfsema (2005) Am J Hum Genet **76,** 572
ex. 14-19dup	Pérez-Grijalba (2019) Mol Genet Genomic Med **7,**

Complex rearrangements: 3 mutations

Description	Reference
Translocation breakpoints within gene	Petrij (1995) Nature **376,** 348
Translocation t(1;16)(p36.2;p13.3)	Kim (2013) Ann Clin Lab Sci **43,** 450
Translocation t(2;16)(q36.3;p13.3)	Petrij (2000) J Med Genet **37,** 168<br>Torres (2010) *Clinics (Sao Paulo)* **65:** 107<br>Rivera (2011) *Clinics (Sao Paulo)* **66:** 1833

Table S2: All of 110 *EP300* mutations causing Rubinstein-Taybi syndrome type 2 listed in HGMDPro variant database and reported in the literature (called the 04/27/2021)

Missense/nonsense: 38 mutations

Nucleotide	Protein	Reference
c.256C>T	p.(Arg86*)	Fergelot (2016) Am J Med Genet A **170,** 3069
c.1092C>A	p.(Cys364*)	Maddirevula (2018) Genet Med **20,** 12<br>Shaheen (2019) *Genet Med* **21:** 545
c.1282C>T	p.(Pro428Ser)	Tamhankar (2015) Indian J Pediatr **83,** 473<br>Negri (2017) *Indian J Pediatr* **84:** 91
c.1833T>G	p.(Tyr611*)	Negri (2016) Hum Mutat **37,** 175
c.1876C>T	p.(Arg626*)	Negri (2016) Hum Mutat **37,** 175
c.1942C>T	p.(Arg648*)	Roelfsema (2005) Am J Hum Genet **76,** 572<br>Xiong (2015) *Science* **347:** 1254806
c.1957C>T	p.(Gln653*)	Cross (2020) Am J Med Genet A **182,** 2508
c.2113C>T	p.(Arg705*)	Fergelot (2016) Am J Med Genet A **170,** 3069
c.2221C>T	p.(Gln741*)	Sequeo (2020) J Med Genet **57,** 760
c.2377C>T	p.(Gln793*)	Negri (2016) Hum Mutat **37,** 175
c.2437C>T	p.(Gln813*)	Negri (2016) Hum Mutat **37,** 175
c.2554C>T	p.(Gln852*)	Fergelot (2016) Am J Med Genet A **170,** 3069
c.3163C>T	p.(Arg1055*)	López (2018) BMC Med Genet **19,** 36
c.3764A>G	p.(His1255Arg)	Welters (2019) Eur J Endocrinol **,**
c.3763C>G	p.(His1255Asp)	Jagla (2017) Clin Dysmorphol **26,** 170
c.3829A>T	p.(Lys1277*)	Negri (2016) Hum Mutat **37,** 175<br>Fergelot (2016) *Am J Med Genet A* **170:** 3069
c.3857A>G	p.(Asn1286Ser)	Fergelot (2016) Am J Med Genet A **170,** 3069<br>Cross (2020) *Am J Med Genet A* **182:** 2508
c.3934C>T	p.(Arg1312*)	Negri (2015) Clin Genet **87,** 148
c.4066C>T	p.(Arg1356*)	Fergelot (2016) Am J Med Genet A **170,** 3069
c.4173G>C	p.(Arg1391Ser)	Cross (2020) Am J Med Genet A **182,** 2508
c.4232C>T	p.(Thr1411Ile)	Negri (2016) Hum Mutat **37,** 175
c.4238T>A	p.(Val1413Asp)	Fergelot (2016) Am J Med Genet A **170,** 3069
c.4301A>G	p.(His1434Arg)	Fergelot (2016) Am J Med Genet A **170,** 3069
c.4363C>T	p.(Gln1455*)	Costain (2018) Eur J Med Genet **61,** 125
c.4390C>T	p.(Gln1464*)	Fergelot (2016) Am J Med Genet A **170,** 3069<br>Cross (2020) *Am J Med Genet A* **182:** 2508
c.4505C>T	p.(Pro1502Leu)	Welters (2019) Eur J Endocrinol **,**
c.4511T>G	p.(Phe1504Cys)	López (2018) BMC Med Genet **19,** 36
c.4585C>T	p.(Arg1529*)	Negri (2016) Hum Mutat **37,** 175
c.4763T>C	p.(Met1588Thr)	Saettini (2018) Pediatr Allergy Immunol **epub,** epub
c.4774A>T	p.(Lys1592*)	Fergelot (2016) Am J Med Genet A **170,** 3069
c.4783T>G	p.(Phe1595Val)	Retterer (2016) Genet Med **18,** 696<br>Hamilton (2016) *Clin Dysmorphol* **25:** 135<br>Costain (2018) *Eur J Med Genet* **61:** 125
c.4933C>T	p.(Arg1645*)	Solomon (2015) Am J Med Genet A **167,** 1111<br>Fergelot (2016) *Am J Med Genet A* **170:** 3069
c.4946G>A	p.(Trp1649*)	Fergelot (2016) Am J Med Genet A **170,** 3069
c.4967T>C	p.(Val1656Ala)	Kosaki (2020) Am J Med Genet A **182,** 1601
c.5471A>C	p.(Gln1824Pro)	Hamilton (2016) Clin Dysmorphol **25,** 135<br>Menke (2018) *Am J Med Genet A* **176:** 862
c.5492G>C	p.(Arg1831Thr)	Liu (2019) N Engl J Med **380,** 2478
c.5506C>T	p.(Gln1836*)	Fergelot (2016) Am J Med Genet A **170,** 3069
c.5824A>T	p.(Met1942Leu)	Wincent (2016) Mol Genet Genomic Med **4,** 39<br>Fergelot (2016) *Am J Med Genet A* **170:** 3069

Small deletions: 35 mutations

Nucleotide	Protein	Reference
c.70_71delTC	p.(Ser24Glyfs*14)	López (2018) BMC Med Genet **19,** 36
c.104_107delCTCT	p.(Ser35Tyrfs*12)	Woods (2014) Am J Med Genet A **164,** 251<br>Fergelot (2016) *Am J Med Genet A* **170:** 3069<br>Boissel (2017) *Genet Med* **:**

c.494_497delTGAA	p.(Met165Thrfs*4)	Bounakis (2015) J Med Case Rep **9,** 10<br>Cross (2020) *Am J Med Genet A* **182:** 2508
c.638delG	p.(Gly213Glufs*7)	Bartsch (2010) Am J Med Genet A **152A,** 181<br>Fergelot (2016) *Am J Med Genet A* **170:** 3069
c.659_662delCTGA	p.(Thr220Serfs*16)	Hamilton (2016) Clin Dysmorphol **25,** 135
c.1140_1143delACAC	p.(His381Alafs*49)	Hu (2018) Genet Med **20,**1045
c.1371_1374delTCAG	p.(Ser457Argfs*7)	Negri (2016) Hum Mutat **37,** 175
c.1407delA	p.(Ala470Leufs*8)	Dillon (2018) Eur J Hum Genet **26,** 644
c.1975_1976delCC	p.(Pro659Lysfs*15)	Fergelot (2016) Am J Med Genet A **170,** 3069
c.2251_2257delTATGGGC	p.(Tyr751Leufs*23)	Fergelot (2016) Am J Med Genet A **170,** 3069<br>Cross (2020) *Am J Med Genet A* **182:** 2508
c.2446delC	p.(His816Thrfs*34)	Masuda (2015) Mol Syndromol **6,** 99
c.2525del	p.(Pro842Leufs* 8)	Sequeo (2020) J Med Genet **57,** 760
c.2877_2884delCACAGAAG	p.(Thr960Glufs*7)	Roelfsema (2005) Am J Hum Genet **76,** 572
c.2966delC	p.(Pro989Glnfs*31)	Negri (2016) Hum Mutat **37,** 175
c.3071_3074delAAGA	p.(Lys1024Argfs*34)	Fergelot (2016) Am J Med Genet A **170,** 3069
c.3093delT	p.(Ser1031Argfs*28)	Luyckx (2019) Eur J Med Genet **62,** 96
c.3234delT	p.(Val1079Trpfs*6)	Fergelot (2016) Am J Med Genet A **170,** 3069
c.3367delC	p.(Met1124Cysfs*33)	Cross (2020) Am J Med Genet A **182,** 2508
c.3438_3439delTG	p.(Glu1147Glyfs*3)	Fergelot (2016) Am J Med Genet A **170,** 3069<br>Cross (2020) *Am J Med Genet A* **182:** 2508
c.3625delC	p.(Gln1209Lysfs*18)	Bartholdi (2007) J Med Genet **44,** 327
c.3661delC	p.(Gln1221Serfs*6)	Hamilton (2016) Clin Dysmorphol **25,** 135<br>Fitzgerald (2015) *Nature***519:** 223<br>Kosmicki (2017) *Nat Genet***49:** 504
c.4078_4086delCTCTTTGCC	p.(Leu1360_Ala1362del)	Fergelot (2016) Am J Med Genet A **170,** 3069
c.4296_4297delAG	p.(Gly1433Alafs*9)	Ishiwa (2019) Pediatr Nephrol **epub,** epub
c.4354_4359delCCTCCT	p.(Pro1452_Pro1453del)	Fergelot (2016) Am J Med Genet A **170,** 3069
c.4371_4376delACCCAA	p.(Ile1457_Lys1459delinsMet)	Fergelot (2016) Am J Med Genet A **170,** 3069
c.4764_4765delGG	p.(Met1588Ilefs*4)	Fergelot (2016) Am J Med Genet A **170,** 3069
c.4783_4784delTT	p.(Phe1595Leufs*19)	Wincent (2016) Mol Genet Genomic Med **4,** 39<br>Fergelot (2016) *Am J Med Genet A* **170:** 3069
c.5578_5584delGGCCAAC	p.(Gly1860Serfs*44)	Cross (2020) Am J Med Genet A **182,** 2508
c.5698_5714del17	p.(Lys1900Aspfs*167)	Hadzsiev (2019) Clin Dysmorphol **28,** 137
c.5873delC	p.(Pro1958Argfs*2)	Hamilton (2016) Clin Dysmorphol **25,** 135
c.6347delC	p.(Pro2116Leufs*18)	Fergelot (2016) Am J Med Genet A **170,** 3069
c.6574_6585del12	p.(Gln2192_Gln2195del)	Fieremans (2016) Hum Mutat **37,** 804
c.6627_6638del12	p.(Asn2209_Gln2213delinsLys)	López (2018) BMC Med Genet **19,** 36
c.7100delC	p.(Pro2367Argfs*36)	Zimmermann (2007) Eur J Hum Genet **15,** 837
c.7222_7223delCA	p.(Gln2408Glufs*39)	López (2016) BMC Med Genet **17,** 97

Small insertions: 13 mutations

**Nucleotide**	**Protein**	**Reference**
c.638dupG	p.(Ser214Lysfs*8)	Baker (2019) J Mol Diagn **21,** 38
c.669dupT	p.(Gln224Serfs*20)	Negri (2015) Clin Genet **87,** 148<br>Fergelot (2016) *Am J Med Genet A* **170:** 3069
c.718dupC	p.(Gln240Profs*4)	Negri (2016) Hum Mutat **37,** 175
c.1167dupA	p.(Val390Serfs*21)	Fergelot (2016) Am J Med Genet A **170,** 3069
c.1554_1555dupAG	p.(Gly519Glufs*3)	Fergelot (2016) Am J Med Genet A **170,** 3069
c.4578_4579insTT	p.(Arg1527Leufs*15)	Fergelot (2016) Am J Med Genet A **170,** 3069
c.4640dupA	p.(Asn1547Lysfs*3)	Negri (2016) Hum Mutat **37,** 175<br>Fergelot (2016) *Am J Med Genet A* **170:** 3069
c.4652dupA	p.(Asn1552Glufs*2)	Negri (2015) Clin Genet **87,** 148
c.4912dupC	p.(His1638Profs*35)	Fergelot (2016) Am J Med Genet A **170,** 3069<br>Fitzgerald (2015) *Nature* **519:** 223<br>Kosmicki (2017) *Nat Genet* **49:** 504
c.4954_4957dupATGT	p.(Cys1653Tyrfs*21)	Fergelot (2016) Am J Med Genet A **170,** 3069<br>McRae (2017) *Nature* **542:** 433
c.5630_5631insGGTA	p.(Thr1878Valfs*6)	Diets (2018) Clin Cancer Res **24,** 1594
c.5723dupC	p.(Thr1909Asnfs*164)	Stavropoulos (2016) NPJ Genom Med **1,** 15012<br>Costain (2018) *Eur J Med Genet* **61:** 125

c.5783dupT	p.(Met1928Ilefs*145)	Jezela-Stanek (2020) Mol Genet Genomic Med **8,** e1263

Small indels: 4 mutations

Nucleotide	Protein)	Reference
c.41_51del11insT	p.(Lys14Ilefs*31)	Negri (2015) Clin Genet **87,** 148
c.1187_1189delCTCinsATT	p.(Ser396_Arg397delinsTyr*)	Negri (2016) Hum Mutat **37,** 175
c.3507_3511delGTTCTinsAG	p.(Phe1170_Ser1171delinsAla)	Fergelot (2016) Am J Med Genet A **170,** 3069
c.1606_1610delins31	p.(Ala536Tyrfs*5)	Cross (2020) Am J Med Genet A **182,** 2508

Splicing: 6 mutations

Nucleotide	Reference
c.1879-12A>G	Negri (2016) Hum Mutat **37,** 175<br>Fergelot (2016) *Am J Med Genet A* **170:** 3069
c.2053+1G>C	Fergelot (2016) Am J Med Genet A **170,** 3069
c.3501+1G>A	Negri (2015) Clin Genet **87,** 148
c.3728+5G>C	Fergelot (2016) Am J Med Genet A **170,** 3069
c.4026-9A>G	Fergelot (2016) Am J Med Genet A **170,** 3069
c.4287-1G>A	Negri (2016) Hum Mutat **37,** 175

Gross deletions: 14 mutations

Description	Reference
2-5 kb, ex. 24-27	Boone (2010) Hum Mutat **31,** 1326
376 kb	Magini (2019) Gene **706,** 162
376 kb, entire gene	Negri (2016) Hum Mutat **37,** 175
4.43 kb incl ex. 24-27	Tsai (2011) Eur J Hum Genet **19,** 43
625 kb incl. entire gene	Novo-Filho (2020) Meta Gene **24** 100702
ex. 1	Roelfsema (2005) Am J Hum Genet **76,** 572
ex. 12-21	López (2018) BMC Med Genet **19,** 36
ex. 17-18	Negri (2015) Clin Genet **87,** 148
ex. 17-19, c.(3142+1_3143-1)_(3590+1_3591-1)del	Negri (2016) Hum Mutat **37,** 175
ex. 24-29, c.(3874+1_3875-1)_(4779+1_4780-1)del	Negri (2016) Hum Mutat **37,** 175
ex. 3-8	Foley (2009) Am J Med Genet A **149A,** 997
ex. 7	Fergelot (2016) Am J Med Genet A **170,** 3069
incl. ex. 20-23	Fergelot (2016) Am J Med Genet A **170,** 3069
incl. ex. 4-8	Cross (2020) Am J Med Genet A **182,** 2508

Table S3: All of 45 *CREBBP* and 8 *EP300* unpublished mutations causing Rubinstein-Taybi syndrome type 1 and type 2 respectively, listed in LOVD database (called the 04/27/2021)

CREBBP mutations: 45

Nucleotide	Protein	Reference
c.(?_-204)_(798-1_799+1)del	p.0?	Martine van Belzen
c.37A>G	p.(Lys13Glu)	Martine van Belzen
c.(85+1_86-1)_(798+1_799-1)del	p.(del)	Martine van Belzen
c.86_233del	p.(Asp29fs)	Johan den Dunnen
c.180_181insTAAA	p.(Pro61*)	VKGL-NL_VUmc
c.181_182insAAGGTTTAA	p.(Pro61delinsGlnGlyLeuThr)	VKGL-NL_VUmc
c.277dup	p.(Ser93Lysfs*19)	Martine van Belzen
c.299del	p.(Gly100Valfs*24)	Mohammed Faruq
c.708dup	p.(Ser237Glnfs*5)	VKGL-NL_Nijmegen
c.881dup	p.(Asn294Lysfs*56)	Johan den Dunnen
c.1011dup	p.(Gln338Thrfs*12)	Martine van Belzen
c.1114C>T	p.(Gln372*)	Martine van Belzen
c.1257G>A	p.(Trp419*)	Mohammed Faruq
c.1388_1395del	p.(Gly463Glufs*7)	Martine van Belzen
c.1412_1415del	p.(Ser471Thrfs*5)	Martine van Belzen
c.1522C>T	p.(Gln508*)	Martine van Belzen
c.1655del	p.(Pro552Argfs*10)	Martine van Belzen
c.1802G>A	p.(Arg601Gln)	Gwenael Le Guyader
c.2283+1G>A	p.?	VKGL-NL_Nijmegen
c.2650_2663dup	p.(Ser889Leufs* 43)	Johan den Dunnen
c.2724del	p.(Ser908Argfs*19)	Martine van Belzen
c.2879del	p.(Pro960Argfs*38)	Martine van Belzen
c.(3060+1_3061-1)_(3698+1_3699-1)del	p.(del)	Martine van Belzen
c.3375T>G	p.(Tyr1125*)	Martine van Belzen
c.3546del	p.(Glu1183Argfs*67)	Martine van Belzen
c.3547G>T	p.(Glu1183*)	Martine van Belzen
c.3610-2A>G	p.(?)	Martine van Belzen
c.3610-1G>A	p.(?)	Martine van Belzen
c.3817_3821dup	p.(Leu1275Ilefs*3)	Martine van Belzen
c.(3836-1_3837-1)_(3982+1_3983-1)del	p.(del)	Martine van Belzen
c.3862_3871del	p.(Arg1288fs)	Martine van Belzen
c.3914+1G>T	p.(?)	Martine van Belzen
c.(3982+1_3983-1)_(*2664_?)del	p.?	Martine van Belzen
c.3993del	p.(Thr1332Glnfs*11)	Mohammed Faruq
c.4134-1G>T	p.?	VKGL-NL_Nijmegen
c.4319_4320del	p.(Phe1440Serfs*12)	Johan den Dunnen
c.4396_4406del	p.(Tyr1466fs)	Martine van Belzen
c.4398dup	p.(Val1467Cysfs*12)	Johan den Dunnen
c.4520T>A	p.(Leu1507Gln)	Xiaochen Qu
c.4559A>G	p.(Lys1520Arg)	Johan den Dunnen
c.4561-2A>G	p.(?)	Martine van Belzen
c.4567_4568del	p.(Phe1523Glnfs*5)	Martine van Belzen
c.4872dup	p.(Met1625Hisfs*35)	Martine van Belzen
c.4898_4908del	p.(Phe1633fs)	Johan den Dunnen
c.5066T>C	p.(Leu1689Pro)	VKGL-NL_Nijmegen
c.5129G>A	p.(Cys1710Tyr)	Johan den Dunnen
c.5223_5224del	p.(Lys1741Asnfs*10)	Johan den Dunnen
c.5641_5642del	p.(Leu1882Alafs*83)	Martine van Belzen
c.5710C>T	p.(Gln1904*)	Martine van Belzen
c.5821C>T	p.(Gln1941*)	Martine van Belzen
c.5843C>T	p.(Pro1948Leu)	Xiaochen Qu
c.6213del	p.(Arg2072Glyfs*3)	Martine van Belzen
c.6324C>G	p.(Tyr2108*)	VKGL-NL_VUmc
c.6436C>T	p.(Gln2146*)	Martine van Belzen
c.6670_6671dup	p.(Met2225Alafs* 78)	Johan den Dunnen

EP300 mutations: 8

Nucleotide	Protein	Reference
c.187C>T	p.(Gln63*)	VKGL-NL_Nijmegen
c.598C>T	p.(Arg200*)	VKGL-NL_Nijmegen
c.2182del	p.(Arg728Glyfs*48)	VKGL-NL_Rot*dam
c.3262del	p.(Asp1088Ilefs*6)	VKGL-NL_Rot*dam
c.3685G>T	p.(Glu1229*)	IMGAG
c.3949G>T	p.(Glu1317*)	VKGL-NL_VUmc
c.4875dup	p.(Gly1626Trpfs*47)	IMGAG
c.5973dup	p.(Gln1992Thrfs*81)	IMGAG
